# Factors Associated with Overweight and Obesity and Their Prevalence Among Medical Students in the Eastern Province of Saudi Arabia: A Cross-Sectional Study

**DOI:** 10.3390/healthcare14142133

**Published:** 2026-07-16

**Authors:** Mothana Al Jabr, Fahad Alanzi, Nouf Alalmaei, Danah Alquwayzani, Saleh Alyousef, Zainab Adel Alali, Sadeem Alkaluf, Abdulrahman Alrashed, Ahmed Alshammari, Azzam Alrashed, Nawaf Alotaibi, Zainab Alasfour, Abdullah Almaqhawi

**Affiliations:** 1 College of Medicine, King Faisal University, Hofuf P.O. Box 400, Al Ahsa, Saudi Arabia; 223004514@student.kfu.edu.sa (F.A.); noufalalmaei@hotmail.com (N.A.); quwayzanii@gmail.com (D.A.); 222418426@student.kfu.edu.sa (S.A.); 224040821@student.kfu.edu.sa (Z.A.A.); 222406242@student.kfu.edu.sa (S.A.); abdulrahman.w.ra@gmail.com (A.A.); 222414172@student.kfu.edu.sa (A.A.); azzam.w.ra@gmail.com (A.A.); 224043364@student.kfu.edu.sa (N.A.); zainabasfour2002@gmail.com (Z.A.); 2 Department of Family and Community Medicine, College of Medicine, King Faisal University, Hofuf P.O. Box 400, Al Ahsa, Saudi Arabia; aalmuqahwi@kfu.edu.sa

**Keywords:** overweight, obesity, medical students, cross-sectional, Saudi

## Abstract

**Background and Objectives:** Overweight and obesity among medical students represent significant public health concerns as unhealthy weight status during medical training may affect future health and professional counseling practices. Understanding the factors associated with BMI categories may help inform future university-based health promotion strategies. This study aimed to assess the prevalence of overweight and obesity and examine sociodemographic, lifestyle, and dietary factors associated with BMI categories among medical students. **Methods:** This cross-sectional study was conducted among medical students at the College of Medicine, King Faisal University, between December 2025 and January 2026. Students were selected using proportionate stratified random sampling according to academic year and gender. Of the 422 students invited to participate, 382 students completed the study questionnaire and anthropometric assessment, yielding a response rate of 90.5%. Height and weight were measured in person using calibrated equipment. Data on demographic characteristics, lifestyle habits, dietary patterns, smoking, sleep, and physical activity were collected through a structured face-to-face questionnaire. Multinomial logistic regression analysis was performed to examine factors associated with BMI categories, using normal weight as the reference category. **Results:** A total of 382 medical students were included, with a median age of 21 years. The prevalence of overweight was 19.9%, and the prevalence of obesity was 14.1%, resulting in a combined overweight and obesity prevalence of 34.0%. In the adjusted multinomial logistic regression model, male gender was associated with being overweight compared with having normal weight (aRRR = 2.71, 95% CI: 1.22–5.98). Obesity showed associations with below-average financial status (aRRR = 13.53, 95% CI: 1.98–92.22), current smoking (aRRR = 17.00, 95% CI: 2.50–115.47), soft drink intake of 2–3 times/week (aRRR = 4.57, 95% CI: 1.13–18.49), and lower physical activity, including no activity (aRRR = 11.61, 95% CI: 2.07–65.01), rare activity (aRRR = 7.54, 95% CI: 2.21–25.80), and physical activity several times/month (aRRR = 5.86, 95% CI: 1.78–19.31). Always eating breakfast was associated with overweight (aRRR = 2.23, 95% CI: 1.05–4.73). Several adjusted estimates had wide confidence intervals, indicating limited precision. **Conclusions:** Overweight and obesity were common among medical students at King Faisal University and were associated with several sociodemographic, lifestyle, and dietary variables. Because of the cross-sectional design and the imprecision of some adjusted estimates, these findings should be interpreted as associations rather than causal relationships. Future longitudinal and multicenter studies using validated lifestyle measures are recommended to clarify temporal relationships and guide university-based health promotion strategies.

## 1. Introduction

Obesity and overweight continue to be major public health problems worldwide, with an increasing burden among adults, adolescents, and young adults. The World Health Organization reported that there were about 2.5 billion overweight adults aged 18 years and older in 2022, including 890 million adults with obesity. This means that 43% of adults globally are overweight and 16% live with obesity [1]. A recent pooled analysis published in The Lancet also demonstrated a substantial rise in the global burden of obesity between 1990 and 2022, with obesity becoming more dominant than underweight in many regions [2]. These trends are concerning due to the strong association between overweight and noncommunicable diseases such as type 2 diabetes, cardiovascular disease, hypertension, dyslipidemia, and several obesity-related complications [1,2,3].

Obesity is a complex chronic disease defined by abnormal or excessive body fat accumulation. While sometimes regarded as a cosmetic issue, it is a serious medical condition associated with an increased risk of multiple comorbidities and adverse health outcomes [4]. According to the World Health Organization (WHO), obesity in adults is defined as a body mass index (BMI)—calculated as weight in kilograms divided by the square of height in meters (kg/m^2^)—greater than or equal to 30 kg/m^2^. Obesity is further categorized into Class I (30.0–34.9 kg/m^2^), Class II (35.0–39.9 kg/m^2^), and Class III (≥40.0 kg/m^2^) [4,5].

Obesity has emerged as a major modifiable risk factor for noncommunicable diseases (NCDs), with its global prevalence is continuing to increase at an alarming rate across both developed and developing regions. Consequences related to obesity and overweight on one’s health have been widely explored in medical literature and are known to account for most deaths and illnesses around the world. Estimates show that overweight and obesity lead to more than 2.8 million deaths and 35.8 million DALYs lost each year [5,6,7].

The Gulf Cooperation Council (GCC), which includes six Arab states on the Arabian Peninsula (Saudi Arabia, Kuwait, Bahrain, Qatar, the United Arab Emirates, and Oman), has a higher prevalence of obesity among the top ten countries in the world, especially among affluent, oil-rich countries. Obesity levels have increased significantly. Among children and adolescents, the prevalence of obesity is about 5–14% in males and 3–18% in females. Among adults, it is seen in about 2–55% of women and 1–30% of men. These changes, alongside urbanization and demographic shifts, are key contributors to the growing obesity burden in the Gulf region [8].

In Saudi Arabia, recent surveys indicate that 28% of men and 44% of women are obese, while 66% of men and 71% of women are either overweight or obese. Similar patterns are observed in Kuwait, where obesity affects 36% of men and 48% of women, and 74% of men and 77% of women are classified as overweight or obese. Overall, obesity prevalence in the Gulf region is consistently higher among women than men [9].

Saudi Arabia has some of the highest rates of NCDs in the world, and the highest in the Arabian Gulf region. Data shows that NCDs cause 73% of all deaths in the country. Cardiovascular diseases cause the most deaths from non-communicable diseases, making up 37% of the total. Cancer follows at 10%, while diabetes and respiratory diseases each account for 3%. Other NCDs make up the remaining 20% [10,11].

Recent studies in Saudi universities have found that lifestyle and psychosocial factors are linked to obesity. University students often experience changes in their eating habits and diets. When students move from high school to university, they often find it hard to keep healthy eating habits. This is usually due to time pressures and stress, which may be related to unhealthy snacking, eating out, and more fast food. These behaviors are major contributors to obesity. For example, among medical students, rates of overweight and obesity have been reported at several universities. For example, at King Khalid University (Aseer), 21.9% of medical students were overweight and 14.6% were obese [12].

Obesity has been widely documented as a burden in Saudi Arabia; however, there are substantial gaps in the literature on medical students in the Al-Ahsa district of the Eastern Province. Regional data show that 26.5% of university students are overweight and 17.1% are obese [13].

While several studies have been conducted in Saudi university and medical students, there is a lack of recent context-specific data from medical students in Al-Ahsa, Eastern Province, particularly studies involving direct anthropometric measurements and detailed assessment of sociodemographic, lifestyle, and dietary factors. Therefore, the present study aimed to estimate the prevalence of overweight and obesity among medical students at King Faisal University using measured height and weight and to explore the sociodemographic, lifestyle, and dietary factors associated with BMI categories. This study provides baseline local evidence based on proportionate stratified random sampling by academic year and gender. It also provides a prevalence estimate and identifies patterns that warrant further investigation in multicenter and longitudinal studies.

## 2. Materials and Methods

### 2.1. Study Design and Setting

The study applied a cross-sectional design to assess the prevalence of overweight and obesity among medical students. It was conducted at the College of Medicine, King Faisal University (KFU), Al-Ahsa, Eastern Province, Saudi Arabia. King Faisal University is viewed as one of the largest universities in the region, with an annual student enrollment exceeding 85,000 students across diverse academic disciplines. The Strengthening of the Reporting of Observational studies in Epidemiology (STROBE) checklist was used to report the study [14].

The study participants were recruited and data was collected between 21 December 2025 and 29 January 2026. The data collection was carried out at allocated sites, which included the College of Medicine classrooms, E-Learning Building, and University Polyclinic, based on the student’s lecture or clinic placement site. Ethical approval was obtained from the King Faisal University Research Ethics Committee (KFU-2025-ETHICS3791). Participation in the study was entirely voluntary, and informed consent was obtained electronically from all participants prior to accessing the survey. Participants were informed about the purpose of the study, the anonymous nature of the questionnaire, and their right to withdraw at any time without consequence. Confidentiality and anonymity were rigorously maintained throughout the research process to protect participants’ identities and ensure compliance with ethical standards.

### 2.2. Study Population and Participants

Participants in the study included 1990 medical undergraduates of the College of Medicine, King Faisal University, comprising 966 males and 1024 females. Lists of students were available from the college administration. These pre-existing administrative records were originally used for academic purposes and were not ordered by student names, academic performance, or demographic characteristics. Each student was assigned a unique serial number in the list, which served as an identifier during sampling. All undergraduate medical students from the first to fifth academic years who were willing to participate and completed the questionnaire were included in the final analysis.

**Figure 1:**
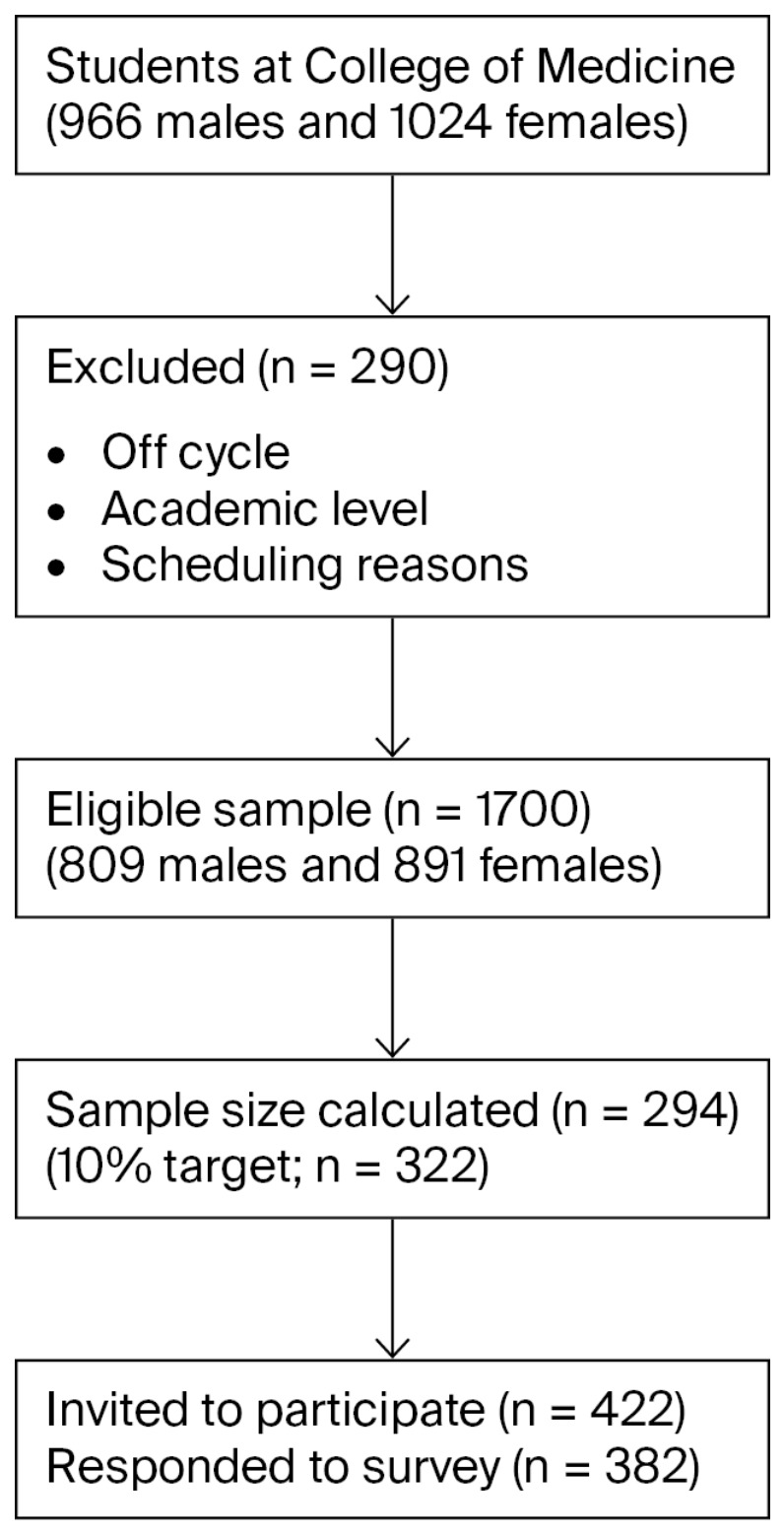
Flowchart of participant selection and inclusion in the study.

Before the final sampling frame was determined, 290 students were excluded. These exclusions included off-cycle students, students with academic interruptions, students not enrolled in the regular first- to fifth-year academic batches during the study period, and students who could not be approached because of scheduling- or placement-related reasons (Figure 1).

### 2.3. Sample Size Determination

**Figure 2:**
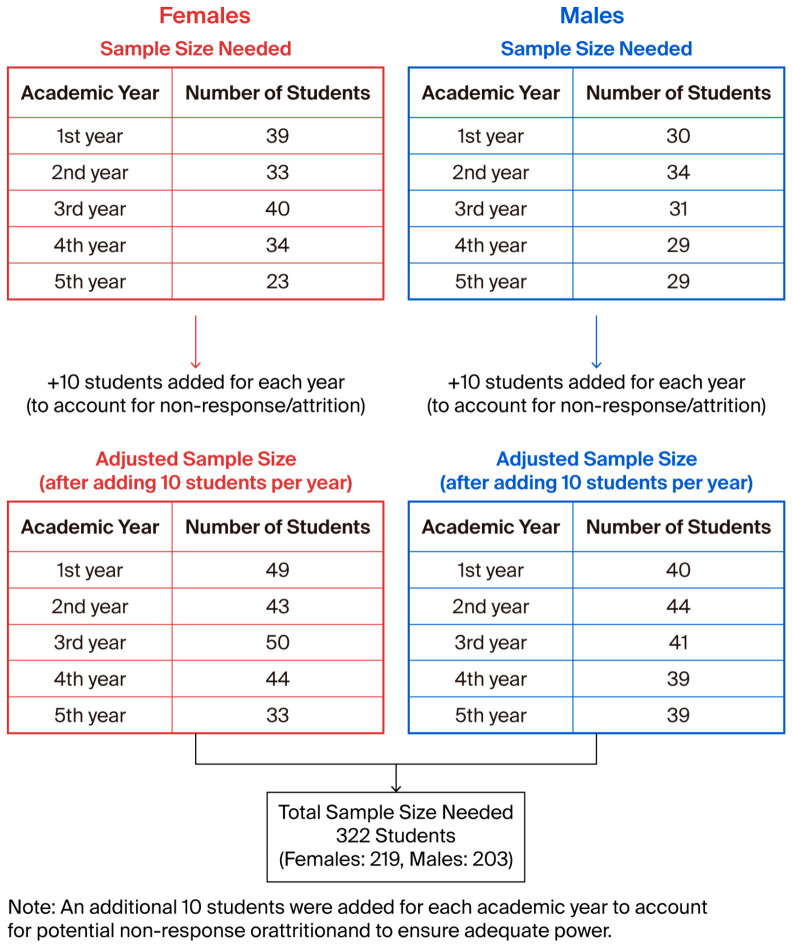
Sample size determination by academic year and gender.

The required sample size was calculated using a prevalence-based sample size formula. An expected prevalence of 36.5% for overweight and obesity among Saudi medical students, based on previously published studies, was used [12]. With a 95% confidence level and a 5% margin of error, the initial sample size was estimated to be 356 students. After applying the finite population correction for the eligible population size (N = 1700), the adjusted sample size was reduced to 294 students. To account for potential non-response, a 10% increase was applied, resulting in a minimum required sample size of approximately 322 students, as shown in Figure 2.

### 2.4. Sampling Technique

A proportionate stratified random sampling method was utilized. Students were stratified into 10 different strata based on academic level (first to fifth year) and gender (male and female). The sample size for each stratum was calculated depending on the size of the stratum to the overall population size.

In the next step, simple random sampling was carried out in each stratum using computer-generated numbers corresponding to the students’ serial numbers. To avoid any issue regarding ineligibility, incomplete data, or non-response, ten more students in each stratum were included in the sample. Thus, the total number of students selected was 422.

Two groups were formed for data collection. One group was assigned to recruit and collect data from male students, while the other was assigned to recruit and collect data from female students. Selected students were contacted by telephone and briefed about the aim of the research. These participants were then called to visit an office in a secluded location.

Anthropometric measurements were taken in person using a standard protocol. Body weight was measured using a calibrated digital weighing scale (Vitafit, China). with participants wearing light clothing and standing barefoot without shoes or bulky items The weighing scale was checked and calibrated before data collection, as per the manufacturer’s instructions. Height was measured using a wall-mounted stadiometer (cm) (Yunseity, China), with the participant standing in bare feet, with their feet together, body erect, and head positioned in the Frankfort horizontal plane. Weight was measured to the nearest 0.1 kg, and height to the nearest 0.1 cm. Data collectors were trained on the standardized measurement procedure, such as position of the participants, use of equipment, and recording of the measurements prior to data collection to minimize inter-observer variability. Afterward, body mass index (BMI) was computed as the weight in kilograms over the height in square meters (kg/m^2^). The BMI classification used was based on WHO guidelines as underweight (BMI < 18.5 kg/m^2^), normal weight (18.5–24.9 kg/m^2^), overweight (25.0–29.9 kg/m^2^), and obese (BMI ≥ 30 kg/m^2^) [1].

Subsequently, a face-to-face survey was conducted to guarantee proper comprehension of the questions and answers. Out of the 423 participants who were invited, 382 successfully took part in the experiment, resulting in a total response rate of 90.3% (Figure 1).

### 2.5. Questionnaire Development

The questionnaire was developed after a thorough review of the previous literature. Several relevant studies were reviewed and served as a foundation for developing the survey questions [5,10,15]. Though validated questionnaires were considered, no validated instrument that covered all domains required to fulfill the objectives of the present study was available. These domains included informed consent, demographic characteristics, lifestyle factors, dietary habits, and dietary consumption patterns of medical students. Therefore, a study-specific questionnaire was developed to ensure that all relevant sections were properly assessed.

Furthermore, a family medicine consultant physician, the study supervisor, and members of the research team added some new questions to increase the relevance and appropriateness of the questionnaire for the target population. A process of refinement was applied to the questionnaire to ensure maximum clarity, relevance, and appropriateness for medical students. A pilot test was conducted with 10 medical students, who did not participate in the final survey. Feedback from the pilot test resulted in improvements to the wording, sequencing, and flow of the questionnaire, which was subsequently revised accordingly. These changes helped ensure that the survey questions were clear and understandable.

Although the questionnaire underwent expert review and pilot testing to improve the clarity, relevance, and content coverage, no formal psychometric validation or reliability testing was performed.

The questionnaire was divided into five separate sections. Section 1 obtained the informed consent to guarantee the free and voluntary participation of the participants in the research. Demographics such as age, gender, academic year, marital status, self-perceived socioeconomic status, and smoking status were discussed in Section 2. Section 3 measured lifestyle factors, including the average number of hours of sleep per night, the amount of time spent using electronic media, such as mobile phones, computers, or television, and levels of physical activity. Section 4 was developed to evaluate eating habits, including regularity of meals (breakfast, lunch, and dinner), late night eating, and eating out behaviors. Section 5 was used to assess dietary consumption patterns, such as fruit and vegetable intake per day, weekly soft drink consumption, and daily fluid intake.

### 2.6. Statistical Analysis

All data analyses were performed using R software (version 4.5.2; R Foundation for Statistical Computing, Vienna, Austria).Continuous variables, which showed a non-normal distribution, were expressed as median and interquartile range (IQR), while categorical variables were expressed as frequency and percentage values. No missing data were identified among the completed questionnaires included in the final analysis.

Body mass index (BMI) was computed using the formula weight/height^2^, where weight is in kilograms and height is in meters and classified based on WHO guidelines as underweight (<18.5 kg/m^2^), normal weight (18.5–24.9 kg/m^2^), overweight (25.0–29.9 kg/m^2^), and obese (≥30 kg/m^2^) [1].

The association between BMI categories and demographic/lifestyle factors was tested using the Chi-square or Fisher’s Exact test where applicable. Differences in continuous variables among BMI categories were tested using the Kruskal–Wallis test.

To identify independent factors associated with BMI categories, multinomial logistic regression was performed with normal weight as the reference category. BMI status was divided into four categories: underweight, normal weight, overweight, and obesity. Therefore, the multinomial logistic regression was used. Normal weight was used as the reference group. This approach enabled the separate estimation of factors associated with underweight, overweight, and obesity compared to normal weight. We did not use binary logistic regression as the primary model because combining overweight and obesity into one outcome would reduce clinical interpretability and may mask differences between factors associated with overweight and those associated with obesity.

Crude relative risk ratios (RRRs) were first estimated, followed by a multivariable adjusted model including variables selected based on evidence from the previous literature, and relevance to the study objectives. Relevant sociodemographic and lifestyle variables were therefore included in the adjusted model. Results were reported as adjusted relative risk ratios (aRRRs) with 95% confidence intervals (CIs).

The variance inflation factors were used to examine the multicollinearity among the independent variables before fitting the multivariable multinomial logistic regression model. Model diagnostics were checked for model stability, sparse categories, and precision of estimates. Sparse categories within variables were interpreted with caution, especially when they were associated with wide confidence intervals. All variance inflation factors were less than five, suggesting no significant multicollinearity.

## 3. Results

### 3.1. Baseline Characteristics

A total of 382 medical students were included in the analysis. The median age was 21 years (IQR: 20–22). Based on BMI classification, 11.8% were underweight, 54.2% had normal weight, 19.9% were overweight, and 14.1% were obese. The combined prevalence of overweight and obesity was 34.0%.

**Figure 3:**
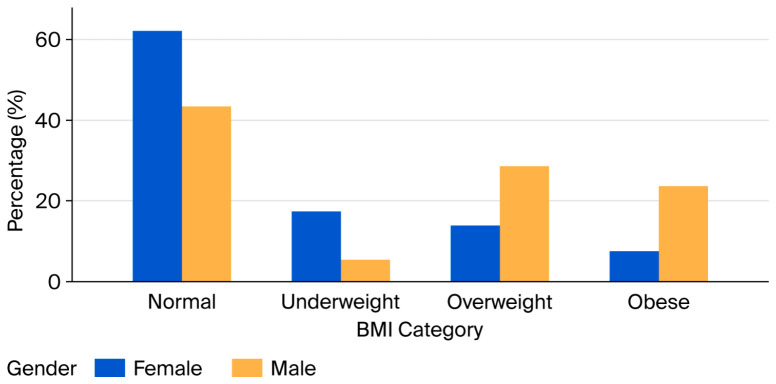
Distribution of BMI categories stratified by gender.

BMI category was significantly associated with gender (*p* < 0.001), smoking status (*p* < 0.001), and financial situation (*p* = 0.028). Male students had higher proportions of overweight (60.5%) and obesity (70.4%) compared to females. No significant associations were observed between BMI and age, marital status, or academic year (Table 1 and Figure 3).

### 3.2. Lifestyle and Dietary Factors

**Figure 4:**
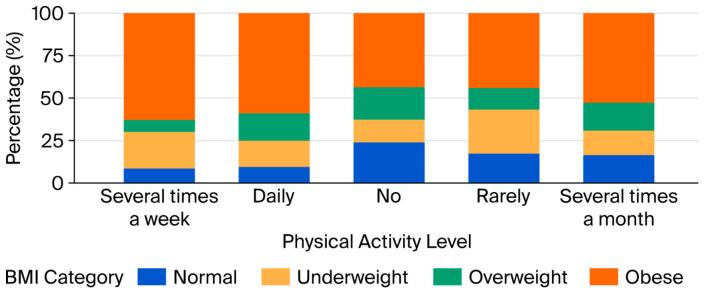
Distribution of BMI categories according to physical activity level.

Significant associations were observed between BMI and lunch intake (*p* = 0.002), vegetable consumption (*p* = 0.010), soft drink intake (*p* = 0.005), fast food consumption (*p* = 0.002), daily water intake (*p* = 0.001), and physical activity level (*p* = 0.034). Higher consumption of soft drinks and fast food was more prevalent among overweight and obese students. No significant associations were found between BMI and sleeping hours, internet usage, breakfast intake, dinner intake, or fruit consumption (Table 2 and Figure 4).

### 3.3. Gender Differences

Several lifestyle and dietary factors differed significantly across gender-specific BMI categories. Significant associations were observed for lunch intake (*p* = 0.002), dinner intake (*p* < 0.001), vegetable consumption (*p* = 0.002), soft drink intake (*p* < 0.001), fast food consumption (*p* < 0.001), daily water intake (*p* < 0.001), and physical activity level (*p* = 0.006). Higher soft drink and fast food consumption were more common among overweight and obese students, particularly males, while lower water intake was more frequent among underweight females. In contrast, sleeping duration (*p* = 0.127), internet usage (*p* = 0.212), breakfast intake (*p* = 0.278), and fruit consumption (*p* = 0.505) were not significantly associated with gender-specific BMI categories. Given the number of comparisons performed in the gender-stratified analysis, the findings in Table S1 should be interpreted as exploratory patterns rather than confirmatory evidence.

### 3.4. Regression Models

**Figure 5:**
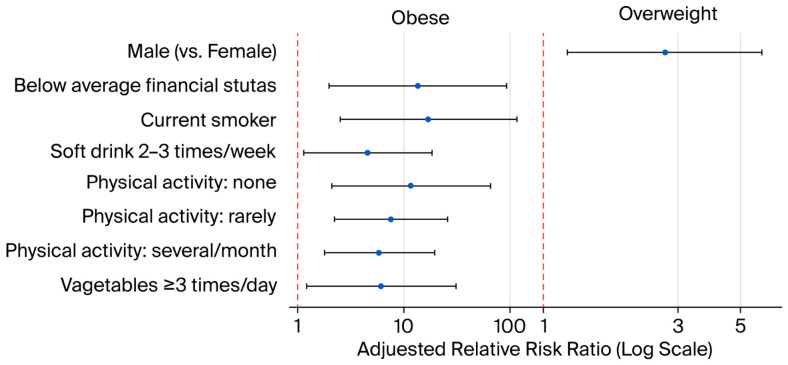
Adjusted multinomial logistic regression for factors associated with BMI categories.

Using normal BMI as the reference category, multinomial logistic regression showed that male students had significantly higher odds of being overweight after adjustment (aRRR = 2.71, 95% CI: 1.22–5.98; *p* = 0.014). Obesity was independently associated with below-average financial status (<5 k SAR) (aRRR = 13.53, 95% CI: 1.98–92.22; *p* = 0.008), current smoking (aRRR = 17.00, 95% CI: 2.50–115.47; *p* = 0.004), soft drink intake of 2–3 times/week (aRRR = 4.57, 95% CI: 1.13–18.49; *p* = 0.033), and lower physical activity (no activity: aRRR = 11.61, *p* = 0.005; rarely: aRRR = 7.54, *p* = 0.001; several times/month: aRRR = 5.86, *p* = 0.004). Always eating breakfast was also independently associated with overweight (aRRR = 2.23, 95% CI: 1.05–4.73; *p* = 0.038). Other sociodemographic and dietary variables were not significantly associated with BMI categories after adjustment (Table 3 and Figure 5).

Several estimates had wide confidence intervals, notably for current smoking and below-average financial status, indicating limited precision that is likely due to small numbers in some exposure categories. Therefore, these estimates should be treated with caution and as exploratory.

## 4. Discussion

Obesity is a major modifiable risk factor for noncommunicable diseases [2]. Its increasing prevalence in young adults is a serious public health concern, given the lasting impact of early lifestyle patterns on future health [3]. However, recent evidence on overweight and obesity among medical students in the Eastern Province of Saudi Arabia is still scarce. Therefore, this study aimed to assess obesity through direct anthropometric measurement to identify the lifestyle correlates associated with excess body weight. Standardized measurements of weight and height were obtained in person, and a structured questionnaire was used to assess demographic and lifestyle factors. The study results show that 34.0% of participants had excess body weight, with 19.9% classified as overweight and 14.1% classified as obese. This burden is clinically relevant because obesity in early adulthood often persists into later adult life [4].

Male students had a significantly higher likelihood of being overweight after adjustment, whereas obesity was independently associated with smoking, low financial status, soft drink intake, and low physical activity. In the univariate analyses, BMI category was also significantly associated with lunch intake, vegetable intake, soft intake, fast food intake, water intake, and physical activity, whereas sleep duration, internet use, and fruit intake were not significant.

However, in the multinomial logistic regression model, after controlling for confounding factors, male sex remained an independent predictor of overweight, and smoking, below-average financial status, soft drink intake, and lower physical activity remained independent predictors of obesity, whereas the other dietary variables did not remain significant. The overall prevalence in our sample is lower than the figures reported in some Saudi university surveys [5], yet it remains substantial for a relatively young and health-literate group.

A study conducted at King Khalid University in southwestern Saudi Arabia reported a combined overweight and obesity prevalence of 36.5% among medical students [5]. Another study at Jazan University showed that 28.3% of medical students had excess body weight [6]. This trend suggests that medical education alone is insufficient to prevent obesogenic practices. The university environment tends to interfere with proper meals and encourage convenient eating habits [7]. Pressure at university can also influence students to opt for high-calorie foods and avoid physical activity [8]. Many studies have found similar high prevalence rates of overweight/obesity among students at universities and other young adults [5,6,9,10]. It is important to note that obesity at an early age is a risk factor which follows one into old age and results in increased burden of disease [4]. In this regard, it is vital to consider the future role of medical students as doctors.

Comparing different studies on obesity among university students is challenging due to differences in sampling methods, BMI assessment, dietary variables, and physical activity definitions. This variability may partly explain the differences in prevalence estimates reported across Saudi and international studies. Another notable finding in our study is that male sex was independently associated with being overweight. This finding is also supported by Alsulami et al., who reported higher obesity-related risk among male university students in Saudi Arabia [10]. The association between male sex and overweight is biologically and behaviorally plausible [10,11].

In regional studies, male students often report higher consumption of fast food, sugary drinks and tobacco than female students [12,13]. Additionally, men may underestimate unhealthy weight gain [16]. Gender norms also influence food choices, leisure patterns and help-seeking behavior [17,18]. In the Saudi and Gulf region, male students are more socially exposed to restaurant food, cafe culture, and smoking environments [19,20]. However, these interpretations should be taken with caution, as the present study did not directly measure gender norms, weight perception, café culture, or detailed social eating environments. These mechanisms could provide context for the association found between male sex and overweight but are not considered as directly demonstrated pathways in the present study. The observed association between smoking and obesity should be interpreted cautiously. Nicotine acutely suppresses appetite [21], but smokers may also have increased central adiposity and poor body fat distribution [22]. Moreover, smoking has been previously associated with insulin resistance, chronic inflammation, and altered lipid metabolism [23,24]. Adolescents who smoke are more likely to engage in other risk behaviors, such as drinking soda, eating dinner late, and being physically inactive [25,26]. However, in the present study, we did not directly measure the intensity of nicotine exposure, central adiposity, insulin resistance, inflammatory markers, lipid metabolism, or behavioral clustering. Hence, the smoking–obesity association should be considered exploratory and interpreted with caution, especially considering the wide confidence interval in the adjusted model. An interesting finding from our study was that perceived poverty was an independent predictor of obesity. This association has been reported previously, suggesting that limited financial resources may reduce access to healthier food options and encourage the consumption of less expensive, energy-dense foods [27]. Although current smoking and below-average financial status were significantly associated with obesity in the adjusted model, the confidence intervals were wide, likely due to the small number of participants in some categories. These findings suggest possible associations but should not be overinterpreted, and confirmation in larger studies is needed.

Food insecurity has been found to be associated with inconsistent eating patterns, compensatory overeating, and reliance on highly processed foods [28,29,30]. Financial hardship could also lead to limited access to places where people do physical activities [31]. Financial stress has been shown to increase stress levels in young adults [32]. However, these explanations should be regarded as potential mechanisms suggested by the previous literature, not findings directly measured in the present study, since household income, food insecurity, food purchasing patterns, psychological stress, and access to physical activity facilities were not assessed. The confidence interval for the association between below-average perceived financial status and obesity was also wide, and therefore, this finding should be interpreted with caution. Size differences, the financial metrics used, and social background can explain some of the differences in the strength of the relationship observed across studies. The nutritional findings of this study are also relevant. A high intake frequency of soft drinks was independently associated with obesity. This is consistent with a systematic review showing an association between sugar-sweetened drinks and obesity [33]. Liquid calories have low satiation and may not induce adequate compensatory responses at subsequent meals [34]. However, total calorie intake, portion size, sugar content, and beverage substitution patterns were not directly measured. Therefore, the soft drink finding should be interpreted as a relationship between the reported frequency of intake and BMI category, not as direct evidence of a measured dietary mechanism. Consumption of fast foods was found to be positively correlated with increased BMI levels. Features of fast foods often involve high energy density, large portion size, refined starch, high sodium content, and sugar-containing drinks [35,36]. Such characteristics could help maintain energy balance despite seemingly unaltered meal frequency. The association reported in this study between always eating breakfast and being overweight should be viewed with caution. This does not necessarily imply that breakfast intake causes obesity. There are several possible explanations, including residual confounding, reverse causality, differences in breakfast composition, greater total daily caloric intake, or reporting bias. Students who were already overweight may have also been more likely to report regular breakfast intake as an attempted lifestyle modification. Future studies are needed to explore this association in a longitudinal design due to the cross-sectional nature of the study and lack of information on breakfast quality and overall caloric intake.

In contrast, some dietary covariates that were significant in the univariate analysis lost their significance upon multivariate adjustment, possibly implying overlap between dietary covariates and other covariates in the multivariate models. The inclusion of a relationship with vegetable intake provides additional information on the dietary covariate pattern among our subjects. An increased intake of vegetables might be an indication of higher diet quality rather than just protection through one single nutrient [37,38]. Diets high in vegetables tend to have lower energy density and a greater satiety effect [39,40]. However, there no relationship was observed between BMI and fruit intake. This may be because of the crude estimation of fruit intake in the questionnaire.

Water consumption was related to BMI categories even in unadjusted analysis, but it was not found to be independently significant. This association may reflect beverage displacement, whereby higher water consumption is associated with lower intake of calorie-containing beverages. Higher consumption of water by students may indicate lower consumption of calorie-containing beverages and snacks [41]. The current literature does not suggest any effect of water consumption on body fatness per se; however, it has been shown that water consumption can displace calorie consumption, thus reducing the amount of adiposity [42]. In our study, this interpretation was further supported by the continued significance of soft consumption in multivariate results.

The lack of physical activity was found to have one of the strongest relationships with obesity. Individuals who were either inactive or engaged in physical activity very infrequently showed significantly increased odds ratios compared to people who engaged in physical activity a few times weekly. This corroborates well with previous studies on the negative impact of sedentary behavior on energy expenditure and metabolic inflexibility [43,44]. Sedentary behavior also results in poor insulin sensitivity and is associated with abdominal fat deposition [45,46]. For medical students, prolonged study sessions and the sedentary nature of academic work are often significant hurdles against engaging in physical activities [47]. Therefore, our findings support the existing body of evidence regarding movement patterns as critical intervention points in preventing obesity in universities.

The relationship between short sleep and obesity is better established in adolescents than in students [48,49]. Sleep duration may not account for other factors, such as sleep quality, circadian type, social jet lag, and nighttime eating, which might be more relevant [50,51]. Internet usage can suffer from the same problem because screen hours may fail to account for simultaneous eating, sedentary behavior, and psychological stress [52,53]. University-based studies have occasionally shown a positive correlation between screen usage and obesity [54,55], but studies on other populations have exhibited attenuation with adjustment for activity and food consumption [56]. Our results indicate that, within our population, food consumption and exercise routines are more directly associated factors of BMI compared to sleep duration and internet usage. Comparisons with the existing literature show general consistency among findings in the Saudi Arabian and Gulf region, although there are differences. In Saudi Arabia, university students consistently exhibit increased BMI in males, along with correlations with sweetened beverages and cigarette consumption [10,57].

Moreover, recent research in Saudi Arabia indicates a connection between obesity and fast-food consumption and lack of physical activity among medical students [5,10]. Analogous findings have emerged regarding rapid nutritional transition and reduction in physical activity among people living in the GCC countries [58,59]. Studies conducted internationally on university students have revealed that high energy intake and sedentary lifestyle are the most prominent predictors of obesity [9]. This may be explained by different approaches adopted by researchers in terms of methodology, sampling, BMI calculation, and control of confounding factors. In this respect, these results are particularly important for medical education and practice in general practice.

Interventions on campus must consider healthier food access, limitations of sugar-laden drinks, and realistic possibilities for organized physical activities. Prevention and treatment of smoking must be included in the health programs for students since smoking is related to obesity within this group. Healthy individuals who practice healthy living may be in a better position to offer counseling on lifestyle choices [60].

These findings may enhance university-based health promotion programs from a public health standpoint. Interventions aimed at increasing physical activity, reducing sugar-sweetened beverage intake, supporting smoking cessation, and promoting healthier dietary practices may improve students’ health. Given that the current findings rely on cross-sectional correlations, future longitudinal and intervention studies are necessary to evaluate the efficacy of these measures.

Strengths of this study include BMI being calculated based on the actual measurement of body parameters rather than reported data about body weight and height, which increased reliability. Proportionate stratified sampling decreased selection bias related to sex and level of education.

However, certain limitations must be considered. Due to the cross-sectional study design, temporal relationships cannot be drawn, which means that it cannot be determined whether these behavioral practices predate obesity. This study was conducted at a single institution, King Faisal University, Al-Ahsa, Eastern Province. Therefore, the findings may not be generalized to all Saudi medical students, students from other regions, or to students in private or differently structured medical colleges. This study only used BMI as a measure of adiposity. BMI is an effective population-level classifier of overweight and obesity, but it does not differentiate between fat mass and lean mass, nor does it measure central adiposity. Additionally, waist circumference, waist-to-height ratio, body fat percentage, and metabolic biomarkers were not assessed. Therefore, BMI misclassification is possible, especially in sex-specific comparisons and in young adults with different body composition profiles. Furthermore, no formal psychometric validation was performed. The findings may be susceptible to recall bias and social desirability bias, as dietary behaviors, physical activity, sleep duration, screen time, smoking status, and eating habits were self-reported. Participants may have underreported unhealthy behaviors or overreported healthy behaviors, which may have influenced the observed relationships. The pilot test only included ten students and was mainly used to improve clarity and flow, so it was not adequate to establish formal reliability or psychometric performance of the questionnaire. Multiple bivariate comparisons were performed without formal adjustment for multiplicity, which increases the possibility of type I error. Hence, we should be cautious in interpreting isolated near-threshold associations. In addition, residual confounding may still be present despite multivariable adjustment, particularly for lifestyle and dietary variables that may not have been fully captured by the questionnaire or measured in this study.

Future research will need to go beyond cross-sectional analysis. It is necessary to conduct longitudinal cohort studies to determine the time frame for the relationship between smoking, drink consumption, sedentary behavior, and weight gain among medical students. Multi-center studies will increase external validity and enable comparisons across different regions in Saudi Arabia. Furthermore, intervention studies can evaluate effective practices, such as alternative beverages, scheduled exercise, and behavioral nutritional counseling.

## 5. Conclusions

Overweight and obese were found to be common among medical students at King Faisal University. Higher BMI categories were associated with several sociodemographic and lifestyle-related factors, including male gender, smoking status, lower perceived financial status, higher soft drink consumption, and lower physical activity. The findings highlight the importance of university-based health promotion strategies targeting modifiable lifestyle behaviors. Because of the cross-sectional design, however, these associations should not be taken as causal. Objective measures of lifestyle in longitudinal and intervention studies are needed in the future to clarify the temporal relationships and to evaluate the effectiveness of targeted prevention strategies.

## Figures and Tables

**Table 1:** Sociodemographic characteristics of medical students according to BMI categories (n = 382).

Variable	Overall (n = 382)	Underweight (n = 45)	Normal (n = 207)	Overweight (n = 76)	Obese (n = 54)	*p*-Value
Gender						<0.001
Female	219 (57.3%)	37 (82.2%)	136 (65.7%)	30 (39.5%)	16 (29.6%)	
Male	163 (42.7%)	8 (17.8%)	71 (34.3%)	46 (60.5%)	38 (70.4%)	
Age (years), median [IQR]	21 (20–22)	21 (20–22)	21 (20–22)	21 (20–22)	21 (20–22)	0.608
Marital status						0.281
Married	23 (6.0%)	5 (11.1%)	13 (6.3%)	4 (5.3%)	1 (1.9%)	
Single	359 (94.0%)	40 (88.9%)	194 (93.7%)	72 (94.7%)	53 (98.1%)	
Academic year						0.345
First	92 (24.1%)	11 (24.4%)	46 (22.2%)	19 (25.0%)	16 (29.6%)	
Second	77 (20.2%)	8 (17.8%)	43 (20.8%)	12 (15.8%)	14 (25.9%)	
Third	83 (21.7%)	11 (24.4%)	39 (18.8%)	25 (32.9%)	8 (14.8%)	
Fourth	83 (21.7%)	10 (22.2%)	49 (23.7%)	15 (19.7%)	9 (16.7%)	
Fifth	47 (12.3%)	5 (11.1%)	30 (14.5%)	5 (6.6%)	7 (13.0%)	
Financial situation						0.028
Less than average	22 (5.8%)	3 (6.7%)	9 (4.3%)	2 (2.6%)	8 (14.8%)	
Average (5–15 k SAR)	256 (67.0%)	31 (68.9%)	133 (64.3%)	55 (72.4%)	37 (68.5%)	
More than average	104 (27.2%)	11 (24.4%)	65 (31.4%)	19 (25.0%)	9 (16.7%)	
Smoking status						<0.001
No	352 (92.1%)	44 (97.8%)	199 (96.1%)	66 (86.8%)	43 (79.6%)	
Past	11 (2.9%)	1 (2.2%)	6 (2.9%)	3 (3.9%)	1 (1.9%)	
Yes	19 (5.0%)	0 (0%)	2 (1.0%)	7 (9.2%)	10 (18.5%)	

**Table 2:** Lifestyle and dietary factors according to BMI categories (n = 382).

Variable	Overall (n = 382)	Underweight (n = 45)	Normal (n = 207)	Overweight (n = 76)	Obese (n = 54)	*p*-Value
Sleeping hours						0.31
<6 h	104 (27.2%)	16 (35.6%)	51 (24.6%)	24 (31.6%)	13 (24.1%)	
6–8 h	242 (63.4%)	26 (57.8%)	139 (67.1%)	45 (59.2%)	32 (59.3%)	
>8 h	36 (9.4%)	3 (6.7%)	17 (8.2%)	7 (9.2%)	9 (16.7%)	
Internet usage (h/day)						0.132
<2 h	16 (4.2%)	0 (0%)	8 (3.9%)	7 (9.2%)	1 (1.9%)	
2–4 h	95 (24.9%)	11 (24.4%)	52 (25.1%)	23 (30.3%)	9 (16.7%)	
4–6 h	150 (39.3%)	19 (42.2%)	76 (36.7%)	29 (38.2%)	26 (48.1%)	
>6 h	121 (31.7%)	15 (33.3%)	71 (34.3%)	17 (22.4%)	18 (33.3%)	
Breakfast intake						0.125
Always	103 (27.0%)	7 (15.6%)	51 (24.6%)	30 (39.5%)	15 (27.8%)	
Sometimes	202 (52.9%)	27 (60.0%)	115 (55.6%)	33 (43.4%)	27 (50.0%)	
Never	77 (20.2%)	11 (24.4%)	41 (19.8%)	13 (17.1%)	12 (22.2%)	
Lunch intake						0.002
Always	263 (68.8%)	23 (51.1%)	143 (69.1%)	52 (68.4%)	45 (83.3%)	
Sometimes	106 (27.7%)	17 (37.8%)	56 (27.1%)	24 (31.6%)	9 (16.7%)	
Never	13 (3.4%)	5 (11.1%)	8 (3.9%)	0 (0%)	0 (0%)	
Dinner intake						0.188
Always	192 (50.3%)	19 (42.2%)	98 (47.3%)	39 (51.3%)	36 (66.7%)	
Sometimes	166 (43.5%)	22 (48.9%)	94 (45.4%)	34 (44.7%)	16 (29.6%)	
Never	24 (6.3%)	4 (8.9%)	15 (7.2%)	3 (3.9%)	2 (3.7%)	
Fruit intake/day						0.573
Zero	151 (39.5%)	20 (44.4%)	76 (36.7%)	32 (42.1%)	23 (42.6%)	
Once	174 (45.5%)	22 (48.9%)	97 (46.9%)	29 (38.2%)	26 (48.1%)	
Twice	44 (11.5%)	3 (6.7%)	25 (12.1%)	12 (15.8%)	4 (7.4%)	
≥3 times	13 (3.4%)	0 (0%)	9 (4.3%)	3 (3.9%)	1 (1.9%)	
Vegetable intake/day						0.010
Zero	105 (27.5%)	18 (40.0%)	53 (25.6%)	17 (22.4%)	17 (31.5%)	
Once	172 (45.0%)	22 (48.9%)	88 (42.5%)	37 (48.7%)	25 (46.3%)	
Twice	72 (18.8%)	5 (11.1%)	49 (23.7%)	15 (19.7%)	3 (5.6%)	
≥3 times	33 (8.6%)	0 (0%)	17 (8.2%)	7 (9.2%)	9 (16.7%)	
Soft drink intake/week						0.005
Zero	105 (27.5%)	17 (37.8%)	64 (30.9%)	17 (22.4%)	7 (13.0%)	
Once	91 (23.8%)	11 (24.4%)	54 (26.1%)	20 (26.3%)	6 (11.1%)	
2–3 times	77 (20.2%)	7 (15.6%)	40 (19.3%)	15 (19.7%)	15 (27.8%)	
>3 times	109 (28.5%)	10 (22.2%)	49 (23.7%)	24 (31.6%)	26 (48.1%)	
Fast food intake/week						0.002
Zero	13 (3.4%)	1 (2.2%)	10 (4.8%)	2 (2.6%)	0 (0%)	
Once	99 (25.9%)	13 (28.9%)	58 (28.0%)	19 (25.0%)	9 (16.7%)	
2–3 times	127 (33.2%)	19 (42.2%)	72 (34.8%)	27 (35.5%)	9 (16.7%)	
>3 times	143 (37.4%)	12 (26.7%)	67 (32.4%)	28 (36.8%)	36 (66.7%)	
Daily water intake (cups)						0.001
<4	71 (18.6%)	18 (40.0%)	43 (20.8%)	6 (7.9%)	4 (7.4%)	
4–6	167 (43.7%)	16 (35.6%)	90 (43.5%)	33 (43.4%)	28 (51.9%)	
7–9	99 (25.9%)	7 (15.6%)	54 (26.1%)	23 (30.3%)	15 (27.8%)	
>9	45 (11.8%)	4 (8.9%)	20 (9.7%)	14 (18.4%)	7 (13.0%)	
Physical activity						0.034
No	37 (9.7%)	7 (15.6%)	16 (7.7%)	5 (6.6%)	9 (16.7%)	
Rarely	86 (22.5%)	10 (22.2%)	38 (18.4%)	23 (30.3%)	15 (27.8%)	
Several times/month	90 (23.6%)	14 (31.1%)	48 (23.2%)	13 (17.1%)	15 (27.8%)	
Several times/week	127 (33.2%)	8 (17.8%)	80 (38.6%)	28 (36.8%)	11 (20.4%)	
Daily	42 (11.0%)	6 (13.3%)	25 (12.1%)	7 (9.2%)	4 (7.4%)	

**Table 3:** Crude and adjusted multinomial logistic regression for factors associated with BMI categories among medical students (n = 382).

Predictor (Reference)	Underweight vs. Normal—Crude RRR (95% CI)	*p*	Underweight vs. Normal—Adjusted aRRR (95% CI)	*p*	Overweight vs. Normal—Crude RRR (95% CI)	*p*	Overweight vs. Normal—Adjusted aRRR (95% CI)	*p*	Obese vs. Normal—Crude RRR (95% CI)	*p*	Obese vs. Normal—Adjusted aRRR (95% CI)	*p*
Gender (female)												
Male	0.41 (0.18–0.94)	0.034	0.65 (0.21–2.02)	0.459	2.94 (1.71–5.05)	<0.001	2.71 (1.22–5.98)	0.014	4.55 (2.37–8.72)	<0.001	1.83 (0.62–5.37)	0.271
Age (per year)	1.04 (0.83–1.30)	0.750	1.87 (0.84–4.20)	0.127	0.90 (0.74–1.08)	0.263	1.01 (0.58–1.74)	0.982	0.91 (0.74–1.13)	0.397	1.09 (0.53–2.24)	0.816
Grade (fifth)												
First	1.43 (0.45–4.54)	0.539	23.25 (0.64–845.67)	0.086	2.48 (0.84–7.35)	0.102	3.47 (0.27–44.82)	0.340	1.49 (0.55–4.05)	0.434	1.69 (0.06–44.29)	0.753
Second	1.12 (0.33–3.75)	0.859	11.25 (0.63–200.34)	0.099	1.67 (0.53–5.25)	0.377	1.76 (0.21–14.82)	0.602	1.40 (0.50–3.87)	0.522	1.58 (0.10–24.30)	0.742
Third	1.69 (0.53–5.39)	0.374	7.96 (0.80–79.72)	0.078	3.85 (1.32–11.23)	0.014	4.29 (0.76–24.31)	0.100	0.88 (0.29–2.70)	0.822	0.93 (0.10–8.56)	0.947
Fourth	1.22 (0.38–3.93)	0.733	3.11 (0.56–17.35)	0.196	1.84 (0.61–5.57)	0.283	1.96 (0.48–7.95)	0.349	0.79 (0.27–2.33)	0.666	0.71 (0.14–3.64)	0.684
Marital status (single)												
Married	1.87 (0.63–5.53)	0.261	2.02 (0.49–8.30)	0.328	0.83 (0.26–2.63)	0.750	0.87 (0.19–3.92)	0.858	0.28 (0.04–2.20)	0.227	0.12 (0.01–1.65)	0.114
Financial situation (average; 5–15 k SAR)												
Less than average	1.43 (0.37–5.59)	0.607	0.56 (0.07–4.22)	0.575	0.54 (0.11–2.57)	0.436	0.52 (0.08–3.26)	0.483	3.20 (1.15–8.86)	0.026	13.53 (1.98–92.22)	0.008
More than average	0.73 (0.34–1.54)	0.402	0.71 (0.29–1.76)	0.456	0.71 (0.39–1.29)	0.257	1.18 (0.57–2.46)	0.653	0.50 (0.23–1.09)	0.082	0.46 (0.16–1.29)	0.141
Smoking (no)												
Past	0.75 (0.09–6.42)	0.796	2.41 (0.21–27.56)	0.479	1.51 (0.37–6.20)	0.569	0.98 (0.18–5.21)	0.980	0.77 (0.09–6.57)	0.812	0.09 (0.00–2.05)	0.132
Yes	0.00 (0.00–∞) *	0.903	0.00 (0.00–0.00) *	<0.001	10.57 (2.14–52.20)	0.004	3.27 (0.53–20.15)	0.202	23.18 (4.90–109.70)	<0.001	17.00 (2.50–115.47)	0.004
Sleep (6–8 h)												
<6 h	1.68 (0.83–3.38)	0.148	1.63 (0.69–3.89)	0.267	1.45 (0.81–2.62)	0.214	1.45 (0.68–3.10)	0.335	1.11 (0.54–2.27)	0.782	0.82 (0.29–2.34)	0.712
>8 h	0.94 (0.26–3.45)	0.930	0.86 (0.20–3.69)	0.834	1.27 (0.50–3.26)	0.617	1.17 (0.34–3.96)	0.805	2.30 (0.94–5.63)	0.068	0.31 (0.06–1.52)	0.148
Internet use (2–4 h/day)												
4–6 h	1.18 (0.52–2.69)	0.690	1.23 (0.45–3.35)	0.682	0.86 (0.45–1.65)	0.657	0.88 (0.40–1.94)	0.749	1.98 (0.86–4.56)	0.110	2.23 (0.70–7.08)	0.172
<2 h	0.00 (0.00–∞) *	0.917	0.00 (0.00–0.00) *	<0.001	1.98 (0.64–6.10)	0.235	2.05 (0.50–8.45)	0.323	0.72 (0.08–6.49)	0.771	0.08 (0.00–2.38)	0.142
>6 h	1.00 (0.42–2.35)	0.998	0.98 (0.34–2.83)	0.977	0.54 (0.26–1.11)	0.096	0.61 (0.25–1.48)	0.272	1.46 (0.61–3.52)	0.393	1.45 (0.43–4.86)	0.550
Breakfast (sometimes)												
Always	0.58 (0.24–1.43)	0.239	0.68 (0.23–1.99)	0.483	2.05 (1.13–3.71)	0.018	2.23 (1.05–4.73)	0.038	1.25 (0.61–2.55)	0.535	1.20 (0.43–3.37)	0.730
Never	1.14 (0.52–2.51)	0.740	0.82 (0.29–2.28)	0.702	1.10 (0.53–2.30)	0.790	1.16 (0.48–2.80)	0.747	1.25 (0.58–2.69)	0.574	1.45 (0.49–4.32)	0.503
Lunch (sometimes)												
Always	0.53 (0.26–1.07)	0.075	0.69 (0.29–1.62)	0.394	0.85 (0.48–1.51)	0.575	0.79 (0.40–1.59)	0.513	1.96 (0.90–4.27)	0.091	1.28 (0.47–3.48)	0.633
Never	2.06 (0.60–7.14)	0.253	2.20 (0.31–15.37)	0.428	0.00 (0.00–∞) *	0.816	0.00 (0.00–0.00) *	<0.001	0.00 (0.00–∞) *	0.832	0.00 (0.00–0.00) *	<0.001
Dinner (sometimes)												
Always	0.83 (0.42–1.63)	0.585	0.73 (0.29–1.83)	0.507	1.10 (0.64–1.89)	0.729	0.74 (0.36–1.53)	0.418	2.16 (1.12–4.15)	0.021	2.41 (0.91–6.37)	0.076
Never	1.14 (0.34–3.77)	0.831	1.36 (0.35–5.37)	0.657	0.55 (0.15–2.03)	0.372	0.93 (0.21–4.14)	0.926	0.78 (0.16–3.76)	0.760	1.64 (0.18–15.09)	0.661
Fruit/day (once)												
≥3 times	0.00 (0.00–∞) *	0.944	0.00 (0.00–0.00) *	<0.001	1.11 (0.28–4.39)	0.876	1.14 (0.21–6.18)	0.878	0.41 (0.05–3.42)	0.414	1.70 (0.12–24.76)	0.697
Twice	0.53 (0.15–1.91)	0.331	0.47 (0.09–2.34)	0.356	1.61 (0.72–3.59)	0.248	1.24 (0.44–3.52)	0.680	0.60 (0.19–1.87)	0.375	0.43 (0.08–2.27)	0.321
Zero	1.16 (0.59–2.28)	0.666	0.73 (0.30–1.79)	0.490	1.41 (0.78–2.53)	0.252	1.76 (0.81–3.81)	0.153	1.13 (0.60–2.13)	0.709	2.00 (0.75–5.34)	0.165
Vegetables/day (once)												
≥3 times	0.00 (0.00–∞) *	0.929	0.00 (0.00–0.00) *	<0.001	0.98 (0.37–2.56)	0.966	0.60 (0.16–2.29)	0.450	1.86 (0.74–4.69)	0.186	6.11 (1.21–30.76)	0.028
Twice	0.41 (0.15–1.15)	0.089	0.37 (0.12–1.16)	0.089	0.73 (0.36–1.46)	0.370	0.53 (0.23–1.23)	0.139	0.22 (0.06–0.75)	0.016	0.18 (0.04–0.84)	0.029
Zero	1.36 (0.67–2.76)	0.398	1.05 (0.41–2.72)	0.915	0.76 (0.39–1.49)	0.427	0.75 (0.32–1.79)	0.520	1.13 (0.56–2.28)	0.735	0.67 (0.24–1.88)	0.444
Soft drinks/week (zero)												
2–3 times	0.66 (0.25–1.73)	0.397	0.55 (0.17–1.75)	0.313	1.41 (0.64–3.14)	0.397	1.31 (0.48–3.52)	0.597	3.43 (1.29–9.14)	0.014	4.57 (1.13–18.49)	0.033
>3 times	0.77 (0.32–1.83)	0.550	0.66 (0.21–2.11)	0.484	1.84 (0.89–3.80)	0.098	1.67 (0.60–4.66)	0.326	4.85 (1.95–12.10)	<0.001	2.59 (0.62–10.79)	0.192
Once	0.77 (0.33–1.78)	0.536	0.54 (0.20–1.48)	0.233	1.39 (0.66–2.93)	0.379	1.48 (0.59–3.69)	0.405	1.02 (0.32–3.21)	0.979	1.14 (0.26–4.96)	0.861
Fast food/week (once)												
2–3 times	1.18 (0.54–2.58)	0.684	1.44 (0.55–3.81)	0.460	1.14 (0.58–2.26)	0.697	1.15 (0.51–2.61)	0.733	0.81 (0.30–2.16)	0.667	0.51 (0.14–1.88)	0.312
>3 times	0.80 (0.34–1.89)	0.609	1.08 (0.34–3.44)	0.900	1.28 (0.65–2.52)	0.483	0.85 (0.33–2.15)	0.724	3.46 (1.54–7.79)	0.003	1.44 (0.43–4.82)	0.551
Zero	0.45 (0.05–3.80)	0.460	0.37 (0.03–4.76)	0.448	0.61 (0.12–3.04)	0.547	0.79 (0.12–5.03)	0.804	0.00 (0.00–∞) *	0.933	0.00 (0.00–0.00) *	<0.001
Water/day (4–6 cups)												
7–9	0.73 (0.28–1.89)	0.514	0.69 (0.23–2.07)	0.507	1.16 (0.62–2.18)	0.641	1.31 (0.60–2.86)	0.490	0.89 (0.44–1.82)	0.755	1.26 (0.47–3.38)	0.651
<4	2.35 (1.10–5.06)	0.028	1.73 (0.67–4.47)	0.255	0.38 (0.15–0.98)	0.045	0.34 (0.11–1.03)	0.056	0.30 (0.10–0.91)	0.033	0.32 (0.07–1.46)	0.142
>9	1.12 (0.34–3.73)	0.847	1.66 (0.40–6.84)	0.483	1.91 (0.87–4.21)	0.109	2.03 (0.77–5.38)	0.154	1.12 (0.43–2.94)	0.810	1.89 (0.50–7.12)	0.345
Physical activity (several times/week)												
Daily	2.40 (0.76–7.58)	0.136	2.19 (0.56–8.62)	0.262	0.80 (0.31–2.05)	0.642	1.03 (0.34–3.11)	0.959	1.16 (0.34–3.98)	0.809	2.67 (0.58–12.26)	0.205
No	4.38 (1.39–13.79)	0.012	2.13 (0.50–9.15)	0.309	0.89 (0.30–2.66)	0.839	2.29 (0.59–8.90)	0.231	4.09 (1.46–11.48)	0.007	11.61 (2.07–65.01)	0.005
Rarely	2.63 (0.96–7.20)	0.060	1.99 (0.62–6.38)	0.249	1.73 (0.88–3.39)	0.111	3.22 (1.36–7.63)	0.008	2.87 (1.20–6.84)	0.017	7.54 (2.21–25.80)	0.001
Several times/month	2.92 (1.14–7.46)	0.026	1.93 (0.62–5.99)	0.256	0.77 (0.37–1.64)	0.502	1.21 (0.49–2.98)	0.682	2.27 (0.97–5.35)	0.060	5.86 (1.78–19.31)	0.004

* Indicates unstable estimates caused by complete or quasi-complete separation (sparse data), resulting in non-estimable relative risk ratios and/or infinite confidence intervals.

## Data Availability

All data are available upon reasonable request from the corresponding author due to restrictions related to subject privacy.

## Supplementary Materials

The following supporting information can be downloaded at: https://www.mdpi.com/article/10.3390/healthcare14142133/s1, Table S1: Distribution of Lifestyle and Dietary Factors Across BMI Categories Stratified by Gender Among Medical Students (n = 382); STROBE Statement—Checklist of items that should be included in reports of cross-sectional studies.
